# Respiratory Care Considerations for Children with Medical Complexity

**DOI:** 10.3390/children4050041

**Published:** 2017-05-19

**Authors:** Jackie Chiang, Reshma Amin

**Affiliations:** 1 Holland Bloorview Kids Rehabilitation Hospital, The University of Toronto, Toronto, ON M4G 1R8, Canada; jchiang@hollandbloorview.ca; 2 Division of Respiratory Medicine, The Hospital for Sick Children, The University of Toronto, Toronto, ON M5G 1X8, Canada

**Keywords:** children with medical complexity (CMC), tracheostomy, ventilation, noninvasive ventilation, polysomnogram, mechanical insufflation-exsufflation

## Abstract

Children with medical complexity (CMC) are a growing population of diagnostically heterogeneous children characterized by chronic conditions affecting multiple organ systems, the use of medical technology at home as well as intensive healthcare service utilization. Many of these children will experience either a respiratory-related complication and/or they will become established on respiratory technology at home during their care trajectory. Therefore, healthcare providers need to be familiar with the respiratory related complications commonly experienced by CMC as well as the indications, technical and safety considerations and potential complications that may arise when caring for CMC using respiratory technology at home. This review will outline the most common respiratory disease manifestations experienced by CMC, and discuss various respiratory-related treatment options that can be considered, including tracheostomy, invasive and non-invasive ventilation, as well as airway clearance techniques. The caregiver requirements associated with caring for CMC using respiratory technology at home will also be reviewed.

## 1. Introduction

Advances in medical care and technology have led to an increasing prevalence of children with medical complexity (CMC) [1]. Children with medical complexity share four key characteristics: (1) chronic health conditions (diagnosed or suspected); (2) severe functional limitations often associated with technology dependence; (3) substantial healthcare needs and (4) high utilization of the healthcare resources [1]. They are deemed the most complex of children with special health care needs (CSHCN), which describes a broad group of children with medical, developmental or psychiatric conditions [2]. Approximately 12–18% of the pediatric population in the United States have been identified as CSHCN; however, there is significant variability in the medical complexity, functional limitations, and resource use amongst CSHCN [1,3].

Although still quite small in absolute numbers (i.e., less than 1% of children), CMC accounts for a large proportion of all childhood healthcare expenditure [4]. They also account for 10% of pediatric hospital admissions and approximately one-quarter will experience readmission within 30 days of discharge [2,5]. Such individuals are at an elevated risk of experiencing adverse health outcomes related to multiple comorbidities, the complicated nature of their care, as well as the frequent interactions with the health system [2]. Common reasons for admission to hospital include major surgery (47%), respiratory tract problems (29%), medical technology malfunction (9%), seizure (6%), and vomiting/feeding difficulties (3%) [6]. Complications account for a significant proportion of the healthcare utilization in addition to morbidity and mortality for CMC [7]. Common respiratory issues identified in CMC include aspiration pneumonia, impaired cough resulting in recurrent respiratory infections, sleep disordered breathing, and respiratory failure. The management of respiratory complications including respiratory technology options for home use will be detailed in this review.

## 2. Respiratory Complications

### 2.1. Sleep Disordered Breathing

Children with medical complexity are predisposed to sleep disordered breathing (SDB) because of their underlying medical conditions affecting the central nervous system, neuromuscular tone and craniofacial structures [2,5] (Table 1).

Sleep disordered breathing is a broad term encompassing abnormalities in respiratory pattern, gas exchange and sleep architecture during sleep [8]. Sleep disordered breathing includes: (1) obstructive sleep apnea (OSA), episodes of complete or partial airway obstruction; (2) central sleep apnea (CSA), prolonged pauses in respiratory effort; and (3) hypoventilation syndrome (HS), persistent low tidal-volume breathing or bradypnea resulting in hypercarbia and hypoxemia [9]. Nocturnal hypoventilation in children is defined as a transcutaneous and/or end tidal carbon dioxide recording of >50 mmHg for >25% of the total sleep time [9].

Obstructive sleep apnea is the most common subtype of SDB in healthy children affecting one to five percent of children [10]. However, all three types of SDB can occur in children with CMC. Sleep disordered breathing has been shown to be up to ten times more prevalent in particular subsets of children with medical complexity (e.g., spina bifida, Chiari malformations, cerebral palsy (CP), neuromuscular disease (NMD)) than in healthy children although the prevalence of SDB in the CMC population overall has yet to be determined [11,12,13,14]. Obstructive sleep apnea is characterized by recurrent periods of increased upper airway resistance with partial or complete intermittent obstruction of the upper airway during sleep. This is usually accompanied by sleep fragmentation and abnormalities in gas exchange. Predisposing factors to the development of OSA include structural and functional factors that increase upper airway collapsibility.

Central sleep apnea is characterized by the absence of central respiratory drive. It may be divided into hypercapneic and hypocapneic causes. Hypercapneic central sleep apneas may be observed in children with central hypoventilation disorders or an underlying neuromuscular cause of hypoventilation resulting in persistently low tidal volumes rather than true apneas [15]. Hypocapneic central sleep apneas are caused by carbon dioxide levels falling below the apneic threshold, which is the carbon dioxide level required to stimulate breathing [15] (Table 1).

Children with nocturnal hypoventilation have elevated carbon dioxide levels, which may be acute or chronic in nature (Table 1). One common contributing factor for hypoventilation in CMC are skeletal abnormalities affecting the thoracic cage resulting in restrictive lung disease characterized by a decrease in total lung capacity (TLC). The most common cause of restrictive lung disease is scoliosis, which not only decreases TLC but also may cause air trapping and subsequent airway obstruction. Individuals with severe scoliosis have higher demands on the functioning rib cage and abdominal muscles in order to maintain adequate tidal volumes for breathing, which may increase the risk of respiratory muscle fatigue and progressive respiratory failure [16]. Children with a measured Cobb angle greater than 90–100 degrees have been found to be at significantly increased risk of chronic respiratory failure and pulmonary hypertension [17].

Children with medical complexity can also progress to having diurnal respiratory failure. This is usually preceded by nocturnal hypoventilation and/or recurrent episodes of pulmonary exacerbations associated with a respiratory infection or aspiration [18]. Chronic respiratory failure may be indicated by hypercapnia defined as arterial carbon dioxide level greater than 45 mmHg (more than 2 standard deviations above the normal independent of child’s age; [19]. The clinical presentation and age of onset of chronic respiratory failure may be highly variable with children presenting after acute respiratory failure or incidentally on a polysomnogram (PSG). The signs and symptoms of SDB as described in healthy children include snoring, pauses in breathing, nocturnal diaphoresis, restless sleep, inattention and hyperactivity, school problems, morning headaches and/or daytime somnolence [10]. Children may also be asymptomatic. However, many of the symptoms of SDB may be difficult to isolate given the medical complexity of these children and their coexistent conditions. In children with NMD, there are established pulmonary function test (PFT) cutoffs that are associated with nocturnal hypoventilation. If a CMC is able to do PFTs, forced vital capacity (FVC) of <60% predicted, and daytime oxygen saturations <93%, may be suggestive of nocturnal hypoventilation [20].

Clinicians caring for CMC need to have a high index of suspicion for SDB based on the underlying medical diagnosis and risk factors because signs and symptoms may be challenging to elicit and certain diagnostic tests may not be feasible. Furthermore, SDB may lead to serious and measurable end-organ dysfunction. The effects of untreated SDB include neurocognitive deficits, cardiovascular complications (including pulmonary hypertension and systemic hypertension), inflammation, growth impairment, reduction in health-related quality of life (HRQOL) and increased healthcare resource utilization [10].

A PSG is the gold standard to diagnose and determine SDB severity. Once a diagnosis of SDB is made, potential treatment options may differ based on the severity and type of SDB as well as the underlying medical condition (Table 2). In healthy children, the most common etiological factor for OSA is adenotonsillar hypertrophy. Adenotonsillectomy is curative more than 70% of the time [10,21]. Adenotonsillectomy is generally considered first line treatment for children with moderate to severe OSA. However, in CMC such as Trisomy 21 or CP, success rates are generally found to be lower [22,23]. For OSA, other surgical interventions may be indicated to relieve the location/cause of the upper airway obstruction (Table 2). In mild OSA, non-surgical interventions include intranasal steroids and leukotriene receptor antagonists, both of which have been shown to reduce upper airway inflammation and improve sleep apnea in children [24,25,26].

Alternative options to treat SDB include noninvasive ventilation (NIV) and tracheostomy with or without mechanical ventilation, with the goals of improving sleep quality, nocturnal gas exchange and reducing the mechanical load on the respiratory muscles [27] (see Section 4.3, Section 4.4 and Section 4.5 for additional details). If SDB is diagnosed, treatment options are often invasive and may not be in the best interests of the child. As a result, outlining treatment options, and understanding a family’s wishes prior to proceeding with a PSG is prudent. Sharing knowledge and decision-making with families may decrease costs to both the child and the healthcare system.

### 2.2. Impaired Cough

Coughing is an important respiratory defense mechanism that clears potential pathogens and maintains airway patency. A typical cough sequentially consists of a full inspiration, closure of the glottis and then contraction of abdominal and expiratory respiratory muscles to generate a positive intrathoracic pressure that forcefully expels air outward after the glottis is opened [28]. Medically complex children may have weakened cough reflexes predominantly due to any underlying condition that results in the absence or reduction in respiratory muscle strength. For example, children with spinal cord injuries may have an inadequate cough due to impairment in the innervations of the diaphragm, intercostal and/or abdominal muscles. Bulbar dysfunction may also result in a weakened cough due to the inability to rapidly open the glottis and maintain patency of the upper airway during coughing [29].

Consequences of an impaired cough include retained secretions and airway debris, which predispose individuals to bronchial mucous plugging. Obstruction of the airways due to mucous and secretions may then lead to microatelectasis causing hypoxemia and hypercapnia, as well as an increased risk of respiratory infections [30]. Over time, recurrent respiratory infections may lead to bronchiectasis due to a destruction of the bronchial ciliated epithelium, muscle layers, and surrounding cartilage. The resulting impaired mucociliary clearance and retention of secretions then further predisposes individuals to chronic bacterial infections and persistent pulmonary inflammation [31]. In addition, impaired respiratory muscle strength leading to diminished cough flows also may result in stiffening of the rib cage and reduction in chest wall compliance further contributing to chronic microatelectasis and overall decreased pulmonary compliance [32].

The strength of a cough can objectively be assessed in those children able to cooperate with the diagnostic testing. A peak flow meter, typically used for asthma, can be used to evaluate cough strength. The child is asked to take a big breath in and then cough into the device. Normal peak cough flow (PCF) values for adults range between 400 to 1200 L/min; peak cough flow values of 270 L/min or less have been shown to increase the risk of pneumonias in adults [32]. In children, peak cough flow rates vary according to gender, height and body surface area [33]. A cutoff of <270 L/min indicating a weak cough is only relevant for children ≥12 years of age as for younger children this may fall within normal range [18,34]. Alternatively, maximal expiratory pressure (MEP), which can be performed in standard pulmonary function laboratories, has been found to correlate with cough strength whereby a MEP of less than 60 cmH_2_O indicates an ineffective cough [35]. MEP testing provides an estimate of expiratory muscle strength at the mouth and is simple to perform in addition to being well tolerated by participants [36]. In contrast to MEP, maximal inspiratory pressure (MIP) and sniff nasal inspiratory pressure (SNIP) evaluate inspiratory muscle strength. During a MIP maneuver, individuals are asked to perform a forceful inspiration against an occluded mouthpiece. SNIP involves a brief sharp voluntary maneuver of inspiration typically through an unoccluded nostril. The disadvantage of such tests is that they are volitional and demand full cooperation in order to obtain accurate results [36].

### 2.3. Aspiration

Aspiration is one of the most common causes of acute hospitalization in CMC [2]. Aspiration is described as the penetration of material below the subglottis and into the lower airways [37]. Acute aspiration events may rapidly progress to pneumonitis, pulmonary edema or even respiratory failure [37]. In contrast, chronic aspiration is characterized by small volume aspiration leading to chronic pulmonary damage, including bronchiectasis and chronic respiratory failure [37]. Children with medical complexity may have two main types of aspiration: (1) aspiration from above (oral secretions or food) or (2) aspiration from below due to gastroesophageal reflux of gastric contents.

#### 2.3.1. Aspiration from Above

##### Aspiration of Oral Secretions

Sialorrhea, or drooling, is frequently observed in CMC. Children with medical complexity are predisposed to sialorrhea often because of comorbid swallowing dysfunction, which can be attributed to a lack of coordinated control of the oropharyngeal musculature, lack of awareness of oral incompetence, impaired sensation to the lower lip or a functional and/or structural inability to close the mouth [38]. Very rarely is sialorrhea actually related to hypersecretion of saliva and is most commonly due to swallowing dysfunction [38]. In children with CP, the prevalence of sialorrhea is estimated to be between 10–58% and is specifically related to oromotor dysfunction, dysphagia and a lack of laryngeal sensation [39].

The treatment of sialorrhea may be multifaceted and can include therapy, medications as well as surgical interventions. In general, treatment approaches follow the conventional paradigm of starting with the least invasive options and escalating the intensity or risk of the intervention as needed. Oral motor skills and sensory awareness can be enhanced through speech therapy programs. The goal of such programs is to improve tongue position and mobility, lip closure, and jaw position and stability [38]. Anticholinergic medications are usually the first line medical treatment of sialorrhea. These include opthalmic atropine drops administered sublingually, glycopyrrolate (usually provided enterally) and transdermal scopolamine patches. The initial agent to be started is influenced by clinician preference, medication cost and insurance plans rather than the literature as to date the superiority of one medication over another has yet to be proven. In one study, comparing the use of atropine, glycopyrrolate and placebo in patients undergoing anaesthesia, the two medications were found to be equally effective for secretion management [40]. The pediatrician needs to be aware of the anticholinergic side effects, which include urinary retention, vomiting, and constipation. Therefore, the general recommendation is to start at low doses and titrate slowly to effect.

Unfortunately, the rate of intolerance to anticholinergics has been reported to be approximately 30% [41]. Special caution should be used with the prescription of anticholinergic agents for children with NMD; the reduction in the saliva may result in thickened secretions and potentially the development of mucus plugs in the airways or tracheostomy tubes (if applicable) resulting in critical airway obstruction [37]. Surgical interventions are usually recommended for children with persistent moderate to severe drooling that has been unresponsive to treatment for at least six months [38]. Botulinum toxin injections may be provided into the submandibular and parotid glands and generally have a lasting effect between six to nine months in duration [42,43,44]. Surgical interventions include salivary duct ligation, ablation and duct removal. General anesthetic is required for Botulinum toxin injections and all surgical interventions. As such, CMC and their families need to be carefully counselled regarding the risks and benefits of the procedure as well as the anesthetic risk. The management of sialorrhea is of particular importance for children being considered for non-invasive positive airway therapy (PAP). See Section 4.5 for more details.

##### Aspiration of Food

Swallowing is a multistep process with both voluntary and involuntary phases. Delayed initiation of swallowing, insufficient laryngeal elevation, and/or impaired opening of the upper esophageal sphincter can all result in aspiration [37]. Although a complete discussion of the diagnosis and management of aspiration from above with food is beyond the scope of this review, it is important to recognize that an aspiration event may bring a CMC into hospital. Additionally, a CMC without a history of aspiration may develop aspiration from above with food in the context of a respiratory exacerbation. Therefore, ongoing assessment of feeding is paramount during a hospital admission when there has been a significant change in the child’s respiratory status. Evaluation of swallowing may take the form of a bedside clinical examination or with more formal diagnostic evaluations such as videofluoroscopy (VFS) or fiberoptic endoscopic evaluation of swallowing (FEES) [37]. If a child is deemed unsafe to feed orally due to signs of aspiration upon a feeding evaluation, alternative options that may be considered include nasogastric tubes, gastrostomy or jejunal tubes, or total parenteral nutrition via intravenous access [7]. Children with NMD are particularly susceptible to feeding difficulties and eventual malnutrition due to progressive oromotor dysfunction, which in turn may further exacerbate respiratory dysfunction [45]. In these situations, gastrostomy insertion has been shown to decrease the frequency of respiratory infections and hospitalization as well as improving overall growth and quality of life [45]. However, anecdotally, parents of CMC often struggle with the decision to completely remove the opportunity for oral feeding as it is often a source of happiness for their child. In these situations, safety must be balanced with the family’s goals of care for their child surrounding quality of life. A taste stimulation program or providing small amounts of thickened feeds or fluids for pleasure could be considered in this context and ongoing assessment by a formal feeding team may be helpful.

#### 2.3.2. Aspiration from Below

Gastroesophageal reflux disease (GERD) is a common problem in CMC and may cause or exacerbate pulmonary disease. Reflux of gastric aspirates into the oropharynx or into the lungs may result in throat irritation, chronic cough, chest congestion and lung inflammation leading to asthma, pneumonia, and even interstitial lung disease [46]. A positive correlation was observed between recurrent pneumonia, abnormal esophageal body function as well as GERD [47]. Investigations for GERD include an upper gastrointestinal series using barium, although reflux cannot be ruled out based on a normal study. Potentially more sensitive measures include gastroesophageal scintigraphy, multichannel intraluminal impedance (MII) pH monitoring, and esophageal pH monitoring, the latter of which is still considered the gold standard for the diagnosis of GERD [46]. Treatment may also include lifestyle changes, such as sleeping with the head of the bed elevated. Medications consist of H2 antagonists or proton pump inhibitors (PPIs), which decrease the acidity of gastric contents. Of note, such medications do not actually decrease the number of reflux events. In addition, there is growing concern that acid suppressive medications may lead to increased bacterial colonization and thus increased susceptibility to infections [48]. Promotility agents may be considered to expedite transit time of digested gastric contents through the GI tract. Surgical options include gastrostomy or jejunostomy tubes and/or fundoplication, which may be performed openly or laparoscopically. However, children with neurologic impairment have been reported to have higher rates of complication and failure with fundoplication [49,50]. Furthermore, fundoplication has not been shown to affect the rate of hospitalization for aspiration pneumonia, apnea, or reflux related symptoms [51].

Children with medical complexity often have several combined risk factors that lead to aspiration [46]. Symptoms suggestive of chronic aspiration include breathing that “sounds wet in nature”, chronic cough, wheezing, choking or gagging with feeds, failure to thrive, and recurrent respiratory infections [52]. In a retrospective study undertaken to identify clinical markers associated with radiographic evidence of oropharyngeal aspiration in children, wet voice, wet breathing and cough were significantly associated with thin fluid aspiration. The most sensitive and specific of the three was wet voice with a sensitivity of 0.67 and specificity of 0.92 [51]. Although evaluation of symptoms is essential, it is also important to note that some children may be asymptomatic but continue to silently aspirate over time. In a prospective study following 300 children with feeding difficulties, oropharyngeal aspiration was detected in 34% of children, 81% of these children were found to silently aspirate. Interestingly, individuals who had silent aspiration were more likely to have neurologic impairment or developmental delay [53]. A low threshold for formal evaluation of aspiration is therefore required for clinicians caring for CMC as it can have a deleterious effect on respiratory health.

## 3. Clinical Cross-Roads: Supporting CMCs and Their Families in the Decision-Making Process to Proceed with Respiratory Technology

The decision to initiate any type of respiratory technology should be accompanied by an informative discussion with the child, family and healthcare team. It is essential that families have a clear understanding of the potential benefits and risks of using the technology in question. In addition, caregiver requirements, financial implications, housing needs, and medical follow up requirements must all be clearly discussed up front. Not only may use of respiratory technology have a significant impact on the child’s quality of life and life expectancy, but it will likely also have a large impact on the entire family’s quality of life, free time, and financial well-being [54].

Clinicians present at these discussions should be those that know the family best and may include the primary care pediatrician, complex care team, respiratory medicine team, additional subspecialists, palliative care team, and social work. It may also be helpful for families to be given an opportunity to connect with other families living with children using similar technologies. Families should be given adequate time to process the information and come to a decision best suited for the patient and family in conjunction with their healthcare team.

Once the decision is made to pursue a particular respiratory technology, ongoing reassessment of the patient’s respiratory status and needs are required. In some individuals, initiation of technology may lead to unexpected or adverse side effects or alternatively, may fail to result in the outcomes expected. Other individuals may require escalating levels of care warranting a change or addition of technology. Throughout this process, it is essential to continue to reassess and discuss the goals of care with patients and their families and recognize that these may change over time.

The discharge process can be tedious and long for CMC on respiratory technology. Ideally, discharge planning should commence as soon as CMC are admitted [7]. The multidisciplinary acute care team should include the primary care pediatrician in the process in addition to partnering with community resources and school services in order to make the transition home as smooth as possible [6]. Prior to discharge, it is useful to ensure that all appropriate vaccinations have been provided. These may include routine vaccinations, annual influenza vaccine and the 23-valent pneumococcal vaccine in those greater than two years of age [55]. In those eligible, organization of respiratory syncytial virus (RSV) prophylaxis should also be considered. Of note, live attenuated vaccines are generally contraindicated in CMC who are taking immunosuppressant medications (e.g., steroids) or who have chronic inflammatory conditions [55].

## 4. Respiratory Technology

### 4.1. Airway Clearance Techniques

Airway clearance techniques (ACT) are used to facilitate removal of secretions from the lung and have been shown to improve sputum expectoration, lung function, symptoms and HRQOL in individuals with non-cystic fibrosis bronchiectasis [56]. Chest physiotherapy in the form of manual percussion is easily administered and can be used to mobilize secretions and remove secretions, often in conjunction with suctioning or postural drainage [57,58]. Other forms of ACT include intrapulmonary percussive ventilation (IPV), which provides high frequency intermittent bursts of air during both the inspiratory and expiratory cycle causing intrapulmonary vibrations that mobilize secretions towards the mouth [18]. In comparison to incentive spirometry (designed to encourage patients to take slow, deep breaths often to prevent microatelectasis in the post-operative period), IPV was shown to have a significant effect on reducing hospitalization days and antibiotic usage in adolescents with NMD [59]. Alternatively, high frequency chest wall oscillation (HFCWO) provides intermittent compression and vibration of the chest wall to produce high frequency, small volume oscillations in the airway using an inflatable vest or jacket to promote secretion clearance [18]. This technique has been shown to improve both pulmonary function and quality of life related parameters in individuals with chronic bronchiectasis [60].

However, in some CMC, the aforementioned ACTs may be ineffective due to the inability to take deep breaths and lack of an effective cough [18]. In such circumstances, cough augmentation devices are likely more effective in clearing secretions, particularly for those with significant neuromuscular weakness. Manual techniques for cough augmentation include glossopharyngeal breathing as well as lung volume recruitment (LVR). Mechanical assisted cough techniques involve the use of mechanical in-exsufflation (MIE) known as “Cough Assist”.

Glossopharyngeal breathing is a technique that can be used to augment the vital capacity. The child voluntarily breaths in sequential boluses of air on inspiration to a maximal tolerated lung volume. Subsequently the child coughs. The augmented vital capacity facilitates a higher peak cough flow. Lung volume recruitment consists of using a self-inflating resuscitation bag attached to a one-way valve. Compression of the LVR bag is coordinated with the patient’s sequential inhalations with the aim of hyperinflating the lungs. This allows for greater peak cough flows than what the patient would be able to achieve independently. Glossopharyngeal breathing as well as LVR may be combined with an abdominal thrust maneuver to further increase peak cough flows [61]. Further potential benefits of glossopharyngeal breathing and LVR relate to the maintenance of chest wall range of motion and lung compliance by preventing atelectasis and contractures of the thoracic cage muscles [62]. However, an important limitation of both of these techniques for the CMC population is that patient cooperation is required. Additional limitations include significant bulbar palsy and ineffective therapy secondary to significant weakness [18]. It is also of paramount importance that patients clearly label the LVR bag so that it is not confused with the self-inflating bag to be used during resuscitation.

Mechanical in-exsufflation can also be administered noninvasively via a face mask or mouthpiece or invasively via tracheostomy. Mechanical in-exsufflation clears respiratory secretions by applying a positive pressure, followed by a rapid shift to negative pressure, thereby simulating a natural cough. If administered via a cuffed tracheostomy, it is recommended to inflate the cuff during use in order to optimize efficacy. Users may preset inspiratory and expiratory pressures as well as duration of inspiratory, expiratory and pause times. Newer models have the added oscillation feature, which is purported to further enhance loosening and mobilization of secretions by adjustments in frequency and amplitude settings. It is important to highlight that MIE does not require patient cooperation. This is a notable benefit over the other techniques. However, there are contraindications to MIE which include: recent hemoptysis, pneumothorax, lung biopsy/lobectomy, history of increased intracranial pressure, cardiac instability, low cardiac output state such as Fontan, nausea or vomiting (Table 3). Complications may include chest pain, muscular stretch or discomfort, bronchospasm/coughing, and gastroesophageal reflux/aspiration and pneumothorax. However, the procedure appears to be well tolerated in children with a stable NMD [63].

Routine use of cough augmentation devices is recommended in CMC deemed to have an inadequate cough, provided no major contraindications are present. A retrospective cohort study performed in adult patients with Duchene Muscular Dystrophy revealed an improvement in the rate of lung function decline (as measured FVC) after initiation of regular LVR [64]. In a retrospective cohort of both children and adults with NMD, routine use of MIE was also found to significantly increase VC by 28% and this remained stable after the second year of use [65]. Furthermore, MIE has been shown to decrease pulmonary complications by reducing the number of respiratory infections, hospitalizations, duration of post-surgical intubation and even mortality when used in combination with NIV [29].

One LVR therapy program suggested, involves eight to ten hyperinflation maneuvers with a five-second breath hold at the end of each maneuver to be performed twice daily [61]. For MIE, general recommendations for routine use include three to five sets of three to five cycles each (of inspiration, exhalation and pause) to be performed twice daily, which may be increased in frequency when unwell. However, the precise prescription for LVR and MIE is usually personalized for the patient. Caregivers must be trained on how to conduct the therapies on these children. In addition, use of pulse oximetry during the therapy as well as having a suction machine at the bedside is recommended during airway clearance sessions.

### 4.2. Oxygen

In healthy term infants, the median baseline saturation during the first year of life is 97–98% and for those one year of age or older, the median is reported to be 98% with a fifth centile of 96–97% [66,67,68]. Children with medical complexity may develop hypoxia requiring the use of home oxygen for a number of different reasons, such as chronic lung disease of prematurity, interstitial lung disease, congenital heart disease, acute lower respiratory tract infections treated at home, or for palliation. Treatment is provided to avert the potential consequences of chronic hypoxemia, which include adverse neurocognitive effects, suboptimal growth, and pulmonary hypertension [69]. However, in children with conditions that may cause hypoventilation or upper airway obstruction, careful monitoring of carbon dioxide levels is necessary to ensure that hypercapnia does not ensue after the initiation of oxygen [69].

The decision to provide oxygen should be undertaken by paediatric specialists with the recommendation that children be assessed (via continuous pulse oximetry rather than arterial blood sampling) for at least 6–12 h, including during sleep and feeding [69]. Home oxygen is typically provided through a low flow system using nasal cannulae, which is the preferred method for children receiving flows of two litres per minute or less [69]. Oxygen equipment most commonly used includes oxygen concentrators with a large cylinder back-up as well as smaller, portable cylinders for travel. Liquid forms of oxygen have limited applications for children and thus are not usually recommended [69]. For CMC requiring high oxygen flows (i.e., ≥4 L/min) or receiving oxygen via tracheostomy, humidification is recommended [69]. Although evidence is lacking with regards to the benefits or harm of routine saturation monitoring using a pulse oximeter, care providers may find it helpful, particularly in the CMC population. In children with respiratory conditions, target oxygen saturation levels of ≥93% have been proposed [69]. However, in children with severe neurodisability and low oxygen saturations, the use of home oxygen should be driven by quality of life issues rather than oxygen saturation targets [69].

Caregivers will require training with respect to how to set up the prescribed oxygen, how to read the flow meter and or oximeter, as well as how to maintain the oxygen equipment with particular awareness of their child`s oxygen prescription, interface, and target oxygen saturations. The discharge destination should have enough space for the oxygen equipment and adequate electricity as well as a functional telephone line. Given that oxygen is highly flammable, caution is required near open flames or generated sparks with smoking, gas cookers, open fires and candles being potential hazards [70]. Smoking in the home should be highly discouraged. A written discharge plan and appropriate follow-up should be arranged prior to discharge [69].

Finally, it is important to recognize that children will need higher oxygen flows during air travel. Commercial aircrafts are now typically pressurized to cabin altitudes of up to 8000 ft (2438 m), resulting in the partial pressure of oxygen equivalent to breathing approximately 15% oxygen at sea level [71]. Altitude exposure may thus worsen hypoxemia in the presence of pulmonary disease. Prior approval by the airline is required if patients choose to take their own oxygen delivery device, which should be in the form of small oxygen cylinders. Of note, only a few oxygen delivery devices are approved for use on airplanes. It is important to review this with the airline in advance. In addition, oxygen may also be supplied by the airline for a fee and must be booked well in advance [71].

### 4.3. Suction Machine

In CMC with significant sialorrhea, bulbar dysfunction or weakened cough causing excess or retained secretions, suctioning will assist in clearing secretions from the naso- and oropharynx and airway. The end goal of suctioning is to prevent airway obstruction from mucous, reduce work of breathing and decrease the risk of atelectasis thus maintaining adequate gas exchange [72].

Suctioning typically involves using a catheter attached to a mechanical suction source to aspirate secretions from either the nares, oropharynx or trachea. Most catheters will have a thumb port that may be occluded to control when suction is applied. Different suction tips will be required for the various methods of suctioning. The procedure should be done as quickly as possible (less than 10–15 s for each pass) with the lowest possible negative pressure and appropriate sized catheter (at a predetermined length) to effectively clear secretions while minimizing the risk of trauma or atelectasis. The usual recommended size of the catheter is no more than half the airway diameter airway [73] and suction pressures for pediatric patients typically range between 80–100 mmHg [74].

In CMC with tracheostomies, a portable suction machine should always be provided as ensuring a patent airway is an essential component of care for a child with a tracheostomy [74]. A clean technique is recommended for suctioning via tracheostomy in the community using a premeasured length [74]. The minimal suction requirements are twice daily (morning and bedtime) to check for patency of the tube in addition to as needed basis when obstruction is suspected, secretions are evident, and before and after a tracheostomy tube change [74,75].

Prior to discharge, care providers should be able to identify when the child needs suctioning and how to use the suction equipment safely, including infection control processes such as how to dispose, clean and replace all materials. Caregivers will also need to be aware of the potential complications of suctioning, such as infection, hypoxia, atelectasis, cardiac dysrhythmias, tracheobronchial trauma and pneumothorax [73,76,77].

### 4.4. Tracheostomy

Children with medical complexity may require tracheostomies for a number of different reasons such as persistent upper airway obstruction, pulmonary hygiene, or long term mechanical ventilation. During surgical tracheostomy tube insertion in the operating room, an artificial opening (i.e., stoma) into the trachea is created to establish an airway through the neck, which is kept patent through the insertion of a tracheostomy tube [75]. The selected tube should extend at least two cm beyond the stoma and no closer than one to two cm to the carina [74]. All tracheostomy tubes have a 15 mm “universal” adaptor to allow bag-mask ventilation in emergency situations [74]. Two of the more commonly used types of pediatric tracheostomy tubes are pediatric Shileys, which are made from polyvinyl chloride (PVC), and pediatric Bivonas, which are silicone based. Uncuffed tracheostomy tubes are preferred over cuffed tubes in the pediatric cohort, as the latter may place excess pressure on the airway epithelium resulting in ischemia and mucosal damage [74]. Currently, there is no consensus with respect to the recommended frequency of tracheostomy tube changes; however, the most common pediatric practice appears to be once weekly changes [74].

Caregivers undergo extensive training prior to taking their child home with a tracheostomy. The training process takes on average between three to four weeks. An alert, awake, tracheostomy trained caregiver is recommended to be with the child with a tracheostomy at all times. This is a very significant caregiver requirement and families must be sufficiently counseled regarding “what life is like to care for a child with a tracheotomy”. Caregivers learn to describe the indications, type and size of their child`s tracheostomy tube as well as the components of the emergency tracheostomy kit (Table 4). They also learn to perform stoma care, suctioning, and tracheostomy changes using a clean (not sterile) technique. Finally, caregivers are trained to be familiar with various emergency procedures, such as manual bagging through a tracheostomy, in the event of accidental decannulation or mucous plugging. Once at home, ongoing evaluation of caregiver competency as well as yearly reassessments of the emergency tracheostomy kit and recertification of cardiopulmonary resuscitation (CPR) is recommended.

Unfortunately, 11–20% of pediatric patients will experience accidental decannulation and/or tracheostomy tube obstruction [78,79,80]. Other acute complications include bleeding, infection, and pneumothorax or pneumomediastinum [81]. Late complications include recurrent tracheitis as well as tracheal stenosis, tracheomalacia and rarely development of a tracheoesophageal fistula [74]. Avoidance of tracheostomy tube obstruction and accidental decannulation are of utmost importance in the care of children with tracheostomies.

### 4.5. Noninvasive Positive Airway Pressure Therapy

Noninvasive PAP is now successfully used in children of all ages and its use is rapidly expanding in CMC [82]. The two main forms of noninvasive PAP utilized in children are (1) continuous positive airway pressure (CPAP) and (2) bilevel positive airway pressure (BPAP). Continuous positive airway pressure provides a single level of positive pressure throughout the respiratory cycle to prevent collapse of the pharynx and increase the cross-sectional area of the upper airway in those with upper airway obstruction [83]. With BPAP use, pressure cycles from a lower expiratory positive airway pressure (EPAP) to a higher inspiratory positive airway pressure (IPAP) when a patient has initiated a breath in order to augment the patient’s own respiratory efforts. BPAP is generally indicated when chronic hypercapneic respiratory failure occurs, abnormalities exist in the control of breathing, or CPAP is inadequate at ameliorating upper airway obstruction and/or is not tolerated [82]. Along with set pressures, adjustments may be made to other settings, such as mode of ventilation, rate, inspiratory time, and trigger sensitivities to improve synchronization between the child and the machine. For children with muscle weakness, it is essential that a backup rate be utilized in the event they are not able to trigger breaths on the machine, particularly during REM sleep when loss of muscle tone occurs [61].

It is important to recognize that not all CMC may be suitable candidates for long term PAP therapy as it is not a secure method of positive pressure therapy and therefore requires a certain degree of respiratory autonomy. Absolute contraindications for use of PAP therapy include haemodynamic instability, pneumothorax, facial trauma or burns, recent upper airway or gastric surgery, loss of gag reflex, and altered level of consciousness [84]. There will be a significant risk of complications in children with insufficient respiratory arousal if the mask is inadvertently displaced and thus children should be able to tolerate periods without PAP. Individuals requiring respiratory support for ≥16 h per day are ineligible largely due to risk of skin breakdown with prolonged use and limitations on development. In addition, in children with severe bulbar palsy, significant sialorrhea or GERD, PAP therapy may be contraindicated due to risk of aspiration. Inability to find an adequate interface or to tolerate PAP therapy may result in avoidance of its use. Lastly, ensuring the competence of caregivers to provide PAP therapy is mandatory. Care providers should receive formalized education on the use of the PAP device assigned to their child. Caregivers also must receive a detailed emergency plan (e.g., what to do in the event of a prolonged power failure), prior to discharge [82].

Noninvasive PAP therapy may be delivered via nasal, oronasal or total face interface. Optimizing interface fit allows for better adherence; however, fewer interface options are available for children in comparison to adults. Approximately half of the failure rates due to poor adherence are attributable to poor interface fit and/or lack of comfort [61]. Complications of use include skin irritation or breakdown, which can be mitigated by adjusting head straps, using protective skin dressings such as duoderm, or alternating different interfaces to provide different pressure points. Drying of the eyes and nasal mucosal can also occur and can be temporized by altering the supplemental humidification level of the machine, using eye lubrication and ensuring a good interface fit to avoid air flow directly into the eyes with nasal and oronasal masks. Over time, ongoing use of PAP therapy may lead to midface hypoplasia, which in turn can itself worsen upper airway obstruction [27] (please see Table 5 for a complete list of complications and strategies). In children that are either not candidates for PAP therapy or intolerant of the therapy, a thoughtful discussion needs to be had with the family and the healthcare team regarding pursuing comfort measures by providing oxygen therapy alone or potentially escalating care via invasive ventilation.

### 4.6. Invasive Ventilation

The number of children requiring long-term invasive ventilation (LTV) at home is on the rise [85]. In the United States, rates of hospital discharges for children receiving LTV have increased from 5026 discharges in 2000, to 7812 discharges in 2006, which is a 55% increase in discharge rates [86]. Indications for invasive ventilation include end state respiratory failure for whom NIV is not an option (due to contraindications or choice), when noninvasive ventilation has failed to control hypoventilation, or based on an emergent need after multiple failed extubations within an acute respiratory failure setting [54]. The goals of such an intervention would be to prevent further deterioration, prolong life, and most importantly improve the quality of life [87].

Initiation of invasive ventilation occurs in the intensive care unit and eligible children must be medically stable with the presence of a stable airway, stable oxygen requirements (usually <40%), and carbon dioxide levels maintained within safe limits on a suitable home ventilator prior to discharge home [88]. Other health conditions should also be well controlled and adequate nutritional intake should be ensured. Furthermore, family members must be willing and capable of meeting their child`s complex care needs in the home environment along with adequate community supports. A minimum of two caregivers willing to be trained should be identified [89]. Suggested training entails both pulmonary and tracheostomy care, ventilator management, infection control practices, suctioning, use of oximetry, provision of medications and oxygen as well as emergency procedures including basic life support certification [89]. Although no validated competency criteria exist for the care of children on home mechanical ventilation (HMV), most programs appear to have similar protocols and include a period of care-by-parent as one of the last steps prior to discharge [54]. Aside from caregiver training, several other necessary components for discharge include (1) suitable nursing support in the community; (2) adequate housing; (3) obtaining all equipment and supplies in the home; (4) school enrollment (if possible); (5) a written discharge care plan and (6) coordination of medical follow-up [54].

Children on HMV, similar to children with tracheostomies, require 24 h per day “eyes on” care due to the significant risk of morbidity and mortality in this population [89]. Their families take on significant healthcare burden as they move into the community and need to be supported in developing the skills required to manage the associated social tensions, emotional burden, financial strain and system navigation required for adaptation to home [90]. Family caregivers undergo an intensive in hospital training period prior to discharge home lasting on average eight weeks. These families require ongoing refresher training, review of emergency tracheostomy kits and annual CPR recertification after discharge.

## 5. Conclusions

Children with medical complexity are at increased risk of developing respiratory problems, which may be related to SDB, impaired cough clearance, and aspiration. The end result of these complications is potential respiratory failure and need for respiratory technology. However, due to the scarcity of empirical evidence specific to CMC, healthcare practitioners often have to extrapolate from other pediatric populations to support their clinical decision making [2]. The main objectives for providing respiratory support should be to optimize quality of life, rehabilitation, growth and development [91].

With continual advances in life sustaining treatments, it is suspected that more CMC will become dependent on respiratory technology in the community. However, the decision to use respiratory technology, particularly LTV, needs to be reached after great thought with respect to the child and family’s best interests and ideally with mutual agreement between the patient, family, and medical team [92]. Children with medical complexity benefit most from the development of interdisciplinary teams—which should include a respiratory specialist—who all work in a coordinated fashion toward shared goals [2].

## Figures and Tables

**Table 1:** Risk factors for sleep disordered breathing (SDB).

Obstructive Sleep Apnea	Central Sleep Apnea	Nocturnal Hypoventilation
Neuromuscular Myopathies (e.g., DMD) Motor neuron disease (e.g., SMA) Spinal cord injury (e.g., cervical spinal cord lesion) Demyelinating disease (e.g., GBS) Airway Abnormalities Laryngomalacia Laryngodystonia Pseudobulbar palsy Vocal cord dysfunction Choanal atresia Micro/retrognanthia Macroglossia Skeletal Abnormalities Scoliosis Kyphosis Thoracic dystrophies Central Causes Arnold Chiari malformations CNS infection CNS tumor CNS stroke/hemorrhage Spinal cord trauma Adenotonsillar Hypertrophy Obesity	Congenital Congenital central hypoventilation syndrome (CCHS) Rapid onset obesity with hypothalamic dysfunction, hypoventilation and autonomic dysregulation (ROHHAD) Arnold Chiari malformations Prader Willi syndrome Joubert syndrome Mobius syndrome Inborn errors of metabolism Neuromuscular disease (NMD) Acquired Central nervous system (CNS) infection CNS tumor CNS stroke/hemorrhage Spinal cord trauma Medications	Neuromuscular Myopathies (e.g., Duchenne muscular dystrophy (DMD)) Motor neuron disease (e.g., Spinal muscular atrophy (SMA)) Spinal cord injury (e.g., cervical spinal cord lesion) Demyelinating disease (e.g., Guillain Barre syndrome (GBS) Skeletal Abnormalities Scoliosis Kyphosis Thoracic dystrophies Pulmonary Parenchymal Disorders Cystic fibrosis Chronic lung disease of infancy Pulmonary hypoplasia

**Table 2:** Treatment options for SDB.

Sleep Disordered Breathing		Causes/Risk Factors
**Obstructive Sleep Apnea**	(1) Mild	Intranasal steroid spray Leukotriene receptor anatagonist
	(2) Moderate to Severe	Surgical Interventions Adenotonsillectomy Revision adenoidectomy Turbinate reduction Genioglossus advancement Midline posterior glossectomy Mandibular distraction Midface distraction Posterior Fossa decompression Tracheostomy Medical Interventions Positive airway pressure therapy (PAP) Weight Loss
**Central Sleep Apnea**		Correct underlying cause if possible Noninvasive ventilation Tracheostomy and invasive ventilation
**Nocturnal Hypoventilation**		Correct underlying cause if possible Noninvasive ventilation Tracheostomy and invasive ventilation

**Table 3:** Contraindications for mechanical in-exsufflation (MIE).

Contraindications	Relative Contraindications
Untreated tension pneumothorax	Emphysematous bullae or subcutaneous emphysema
Active hemorrhage with hemodynamic instability (including pulmonary hemorrhage)	Recent epidural spinal infusion or spinal anaesthesia
Suspected or confirmed head and/or c-spine injury	Burns, open wound, infection or skin grafts on the thorax or the face
Unrepaired tracheoesophageal fistula	Recently placed transvenous pacemaker or subcutaneous pacemaker
Uncontrolled asthma or bronchospasm	Suspected pulmonary tuberculosis
	Pneumothorax
	Select airway anomalies (e.g., tracheobronchomalacia)
	Recent barotrauma
	Recent lobectomy/pneumonectomy
	Severe obstructive lung disease (e.g., severe asthma)
	Cardiac instability where small intrathoracic pressure changes may affect cardiac output (e.g., Fontan circulation)—with cardiology approval prior to initiation
	Evidence of increased intracranial pressure (ICP) or external ventricular drain (EVD)—with neurosurgical approval prior to initiation given the potential risk of in-exsufflation therapy increasing ICP
	Known susceptibility to pneumothorax/pneumomediastinum or previous pneumothorax/pneumomediastinum
	Nausea and vomiting
	Infants less than 3 months of age

**Table 4:** Contents of an emergency tracheostomy kit.

**Emergency Tracheostomy Kit**
(1) Tracheostomy tube of the same size used with obturator (attached to tracheostomy ties) (2) Tracheostomy tube 1/2 size smaller (3) Pre-cut tracheostomy gauze (4) Scissors (5) Normal saline (6) Lubricant (7) Feeding catheter attached to syringe for manual suction
**Additional Equipment**
(1) Emergency contact list (2) Heat and moisture exchangers (HMEs) (3) Oximeter (4) Manual resuscitation bag (5) Portable suction machine, tubing and catheters (6) External batteries for equipment (7) Oxygen (if applicable) (8) Compressor (if applicable)

**Table 5:** PAP complications and corresponding strategies.

Complications	Strategies
Skin erythema and breakdown	Ensure proper fit Alternate interfaces Alternate interface compositions (e.g., gel, air) Use of protective dressing and gel pads
Midface hypoplasia	Titrate pressure to minimum effective pressure Maximize time off PAP Routine evaluation of maxillomandibular growth
Gastric insufflations and aspiration	Avoid PAP if ongoing emesis Vent Gastrostomy tube Optimize GERD management Monitor closely when PAP first introduced with concurrent feeds
Nasal congestion and epistaxis	Use supplemental humidification Consider nasal steroids for congestion Consider change from nasal to oronasal mask with intercurrent illness
Eye irritation	Ensure proper mask fit Use artificial tears
Rebreathing carbon dioxide	Ensure smallest and best fitting mask used Clinically assess oronasal and total face mask before discharge
Pulmonary air leak	Admit patient to hospital: decision to hold PAP or decrease pressure should be made on a case by case basis Titrate pressure to minimum effective pressure
Cardiovascular complications	Caution in children with single ventricles or hypovolemic states

GERD: Gastroesophageal Reflux Disease; Adapted from [65].
